# Altered detrusor contractility and voiding patterns in mice lacking the mechanosensitive TREK-1 channel

**DOI:** 10.1186/s12894-019-0475-3

**Published:** 2019-05-21

**Authors:** Ricardo H. Pineda, Joseph Hypolite, Sanghee Lee, Alonso Carrasco, Nao Iguchi, Randall B. Meacham, Anna P. Malykhina

**Affiliations:** 1 Division of Urology, Department of Surgery, University of Colorado Denver,Anschutz Medical Campus, 12700 E 19th Ave, M/S C317, Aurora, CO 80045 USA; 2 Department of Urology, University of California San Diego, 3855 Health Science Drive, Room 4345, Bay 4LL, La Jolla, CA 92093 USA; 3 Children’s Mercy Hospital, 2401 Gillham Rd, Kansas City, MO 64108 USA; 4 Division of Urology, Department of Surgery, University of Colorado Denver, Academic Office One Bldg., Rm 5602, 12631 East 17th Ave., M/S C319, Aurora, CO 80045 USA

**Keywords:** Urinary bladder, TREK-1 channel, Detrusor mechanosensitivity, Voiding, Micturition cycle

## Abstract

**Background:**

Previously published results from our laboratory identified a mechano-gated two-pore domain potassium channel, TREK-1, as a main mechanosensor in the smooth muscle of the human urinary bladder. One of the limitations of in vitro experiments on isolated human detrusor included inability to evaluate in vivo effects of TREK-1 on voiding function, as the channel is also expressed in the nervous system, and may modulate micturition via neural pathways. Therefore, in the present study, we aimed to assess the role of TREK-1 channel in bladder function and voiding patterns in vivo by using TREK-1 knockout (KO) mice.

**Methods:**

Adult C57BL/6 J wild-type (WT, *N* = 32) and TREK-1 KO (*N* = 33) mice were used in this study. The overall phenotype and bladder function were evaluated by gene and protein expression of TREK-1 channel, in vitro contractile experiments using detrusor strips in response to stretch and pharmacological stimuli, and cystometry in unanesthetized animals.

**Results:**

TREK-1 KO animals had an elevated basal muscle tone and enhanced spontaneous activity in the detrusor without detectable changes in bladder morphology/histology. Stretch applied to isolated detrusor strips increased the amplitude of spontaneous contractions by 109% in the TREK-1 KO group in contrast to a 61% increase in WT mice (*p* ≤ 0.05 to respective baseline for each group). The detrusor strips from TREK-1 KO mice also generated more contractile force in response to electric field stimulation and high potassium concentration in comparison to WT group (p ≤ 0.05 for both tests). However, cystometric recordings from TREK-1 KO mice revealed a significant increase in the duration of the intermicturition interval, enhanced bladder capacity and increased number of non-voiding contractions in comparison to WT mice.

**Conclusions:**

Our results provide evidence that global down-regulation of TREK-1 channels has dual effects on detrusor contractility and micturition patterns in vivo. The observed differences are likely due to expression of TREK-1 channel not only in detrusor myocytes but also in afferent and efferent neural pathways involved in regulation of micturition which may underly the “mixed” voiding phenotype in TREK-1 KO mice.

## Background

The urinary bladder undergoes slow mechanical stretch during the storage phase of the micturition cycle without significant changes in intravesical pressure [5, 29]. Previous animal studies provided evidence that bladder stretch can activate mechanosensitive two-pore domain potassium (K_2P_) channels [29, 46, 49]. The family of mechano-gated K_2P_ channels is highly expressed in the smooth muscle of visceral hollow organs where they regulate smooth muscle excitability by controlling the resting membrane potential [3, 4, 29, 52, 53]. Our previous studies confirmed that bladder capacity and detrusor relaxation in the human urinary bladder depends on the expression and function of TREK-1 channel, one of the members of the K_2P_ channel family [34]. TREK-1 has also been detected in the human myometrium, where it participates in the maintenance of uterine relaxation during pregnancy [3, 10, 55]. Further, expression of TREK-1 channels in vascular smooth muscle suggests a role in the regulation of the vascular tone and endothelial production of nitric oxide [44, 45].

A decrease in functional expression of TREK-1 channel in the bladder smooth muscle was shown to be associated with detrusor overactivity (DO) in the animal model of partial bladder outlet obstruction [4]. In humans, increased expression of TREK-1 was detected in pregnant women’s myometrium. Interestingly, TREK-1 expression levels declined by the time of labor [3, 10, 55]. Experimental data obtained by our group in bladder specimens from patients with idiopathic DO confirmed a decreased expression of TREK-1 channels along with an altered response to pharmacological stimulation and diminished smooth muscle relaxation. Additionally, DO specimens had an increased basal tone and increased spontaneous contractile activity suggestive that TREK-1 channels affect bladder compliance during storage phase of the micturition cycle [48].

Limitations of in vitro experiments on isolated human bladders included the inability to assess the full spectrum of TREK-1 related effects on voiding function as the channel is also expressed in the nervous system and therefore, may indirectly participate in micturition modulation via a neural pathway. Members of the mechanosensitive K_2P_ channel family are abundantly expressed in the brain and sensory neurons of the dorsal root ganglia in humans [18, 20, 41]. However, the studies of functional changes in human neurons and nerve fibers have several ethical challenges making the use of animal models necessary for this type of investigations. Additionally, the absence of TREK-1 selective activators and inhibitors makes it difficult to isolate TREK-1 from other K_2P_ channels in primary cultured cells. Therefore, in the present study, we aimed to evaluate the effects of TREK-1 gene knockout on bladder contractile function and voiding patterns by comparing wild-type (WT) and TREK-1 KO mice. We comprehensively assessed bladder phenotype in vivo and in vitro*,* measured detrusor basal tone, spontaneous activity, contractile function and relaxation in response to nerve and muscle-mediated stimulation of bladder smooth muscle (BSM), as well as determined bladder responses to different pharmacological stimuli.

## Methods

### Animals and experimental groups

Adult male and female C57BL/6 J wild-type (WT, *N* = 32) and TREK-1 knockout (*Kcnk2*^*−/−*^, TREK-1 KO, *N* = 33) mice were used in this study. Wild type mice (10–12 weeks old) were obtained from Jackson Laboratories (Bar Harbor, ME, USA). TREK-1 KO mice, a generous gift from the laboratory of Dr. Min Zhou (Department of Neuroscience, The Ohio State University, Columbus, OH), were bred in the Breeding Core Barrier Facility at the University of Colorado Anschutz Medical Campus. Originally, the *Kcnk2* ^−/−^ mice were generated as described in [45]. Briefly, the TREK-1 gene, *Kcnk2*, spans approximately 136 kbp of the *M. musculus* genome and includes eight exons. The knockout mice were generated by replacing 4 kbp of the *Kcnk2* gene including the second exon (excluding the first 23 bp), the second intron, the third exon and the first 23 bp of the third intron with a β-galactosidase/Neomycin selection cassette producing a truncated, nonfunctional protein.

All animals were housed in a regulated environment on a 12:12-h light/dark cycle with free access to food and water. There were no overt differences in feeding behavior, litter size, growth rate and body weight between the WT and TREK1 KO mice. All protocols were approved by the University of Colorado Institutional Animal Care and Use Committee.

### RT-PCR for genotyping and PKC isoform expression

Genomic DNA was extracted using the REDExtract-N-Amp Tissue PCR Kit (Sigma-Aldrich, St Louis, MO. USA) from the urinary bladders of WT and TREK-1 KO mice. Genotyping was performed as previously described [45]. Briefly, the WT *Kcnk2* allele was detected by using the following primer pairs: forward 5′-GCTGGGTGAAGTTCTTCAGC-3′, and reverse: 5′- CATTACCTGGATGAGTTCGTC-3′. The *Kcnk2* KO allele was detected using the primer pairs: forward 5′-GCAGCGCATCGCCTTCTATC-3′ and reverse 5′-AGGAGATGAAGACCTCTGCAAAGG-3′. End-point RT-PCR products from WT and TREK-1 KO specimens were subsequently run on 2% agarose gels. Gel images were taken and analyzed using the Doc-It LS Image Acquisition & Analysis System (UVP, LLC, Upland, CA, USA).

To compare the gene expression levels of several protein kinase C (PKC) isoforms expressed in the bladder, whole bladder tissues were removed from WT and TREK-1 KO mice, and snap-frozen in liquid nitrogen. Posteriorly, total RNA was extracted and purified using TRIzol® Plus RNA Purification Kit (Ambion, Thermo Fisher Sci. Waltham, MA, USA) according to the manufacturer’s instructions. RNA concentrations and purity were determined spectrophotometrically on a Nanodrop device (Thermo Fisher Sci. Waltham, MA, USA). RNA integrity was evaluated by formaldehyde agarose gel electrophoresis, stained with ethidium bromide, and visualized under UV light. Isolated RNAs were stored at − 80 °C until use. First-strand complementary DNA (cDNA) was synthesized using the Qiagen OneStep RT-PCR kit (Qiagen, Valentia, CA. USA) by the manufacturer’s guidelines. Products were stored at − 20 °C until the use. End-point PCR was performed using the Qiagen Multiplex PCR plus kit (Qiagen, Valentia, CA. USA) following the manufacturer’s instructions. The following PKC isoforms were selected for comparison due to their significant level of expression in the urinary bladder: PKC-α (alpha), PKC-β (beta), PKC-γ (gamma), PKC- δ (delta), PKC-ε (epsilon), PKC-μ (mu) and PKC-τ (tau). Primer sequences for each isoform, gene accession numbers and predicted RT-PCR product sizes are listed in Table 1. Non-template control reactions were included in each reaction to test for possible RT-PCR contamination and were run in parallel with the experimental samples. End-point PCR products were analyzed by electrophoresis in 1.5% agarose gel, stained with ethidium bromide, and visualized under UV light.

**Table 1:** Primers for PKC isoforms expressed in the urinary bladder

Name	Sequence	Fragment Size (bp)	Accession Number
PKCα-F	AGGAGCCACAAGCAGTATTC	93	NM_011101.3
PKCα-R	CCAGCTTCAGATCCCTGTAAAT		
PKCβ-F	GCAGAGCAAGGGCATTATTTAC	114	NM_001316672.1
PKCβ-R	CCATCCCAGATGTTCTCCTTAC		
PKCγ-F	GCACCTGAGATCATTGCCTATC	90	NM_011102.4
PKCγ-R	CTGTCCTGCCAACATCTCATAC		
PKCδ-F	TAGTGAGGAGGAGGCAAAGT	105	NM_001310682.1
PKCδ-R	CCGAAGAAGGTGGCGATAAA		
PKCε-F	GCTCGGAAACACCCTTATCT	100	NM_001310682.1
PKCε-R	ACATGAGGTCTCCACCATTTAC		
PKCμ-F	GCAGTGGAGTTAGAAGGAGAAG	123	XM_006515590.3
PKCμ-R	GGCTCACAGGAGACAGTAAAG		
PKCτ-F	CAGGGACCTGAAGCTTGATAAT	96	NM_008859.2
PKCτ-R	GCATCTCCTAGCATGTTCTCTT		

### Western blotting

Total protein was isolated from the urinary bladder by conventional tissue lysis with T-PER Tissue Protein Extraction Reagent (ThermoFisher Sci, Waltham, MA. USA) containing protease and phosphatase inhibitors (Sigma Aldrich, St Louis, MO. USA). Western blot experiments were performed with equal loads of the total protein per lane (20 μg). WT and KO samples were size fractionated by SDS-PAGE electrophoresis using 4–10% polyacrylamide Mini Protein gels (BioRad, Hercules, CA. USA). Proteins were then electrotransferred to polyvinylidene difluoride membranes (LICOR Biotechnology, Lincoln, NW. USA) and incubated for one hour at room temperature (RT) in Odyssey Blocking Buffer (TBS, LICOR Biotechnology, Lincoln, NW. USA). Membranes were subsequently washed three times in PBST for 10 min each and incubated with Anti-TREK-1 (F6, 1:1000, Santa Cruz Biotechnology, Dallas, TX. USA) and anti-Histone H3 as a house-keeping gene (1:2000, Abcam, Cambridge, MA. USA) in blocking buffer under constant agitation. We also tested other commercially available antibodies from the same company which included the C20, E-19, and H-75 anti-TREK-1 variants. The C20 and E19 anti-TREK-1 antibodies did not work for Western blotting, while H-75 antibody (aa352–426 within the C-terminus) showed two bands in WT animals and one band in TREK-1 KO (data not shown). Therefore, we used F6 anti-TREK-1 for Western blotting. The next day, membranes were rinsed three times with PBST for 10 min each time, and incubated with anti-mouse and anti-rabbit VRDye secondary antibodies (1:10000, LICOR Biotechnology, Lincoln, NW. USA) for 2 hrs at RT. Afterwards, membranes were rinsed three times with PBST for 10 min each and imaged using the Odyssey CLx imaging system (LICOR Biotechnology, Lincoln, NW. USA).

### Tissue immunohistolabeling

WT and TREK-1 KO animals were anesthetized with isofluorane (2.5%) and perfused via the left cardiac ventricle with phosphate-buffered-saline (PBS, pH 7.4) followed by 4% paraformaldehyde in PBS (PFA) for 10 min. The colon, bladder, and kidneys were removed and placed in PFA for two hours at 4^o^ C. Subsequently; the tissues were placed in 70% alcohol for at least 2 days before being dehydrated and embedded in paraffin. 4 μm -thick sections were cut and mounted on “frosted” slides (Fisher Scientific Co, Waltham, MA. USA) at the Morphology and Phenotypic Core of the University of Colorado Anschutz Medical Campus. Before immunolabeling, sections were washed in Xylene (Sigma-Aldrich, St Louis, MO. USA) to remove paraffin, re-hydrated in a graded ethanol series, washed with distilled water, and rinsed in 50 mM Tris-buffered saline (TBS, pH 7.4) for 5 min. Heat-induced epitope retrieval was performed by incubating the slides at 85–90^0^ C for 15 min in Target Retrieval Solution (DAKO, Agilent Pathology Solutions, Santa Clara, CA. USA). Once cold down to room temperature, the sections were washed twice in TBS containing 0.1% Tween 20 (TBST, Sigma-Aldrich, St Louis, MO. USA), and incubated for 30 min in casein blocking solution (CBS, DAKO, Agilent Pathology Solutions, Santa Clara, CA. USA). Slides were incubated overnight at 4^0^ C (ON) in CBS with mouse monoclonal antibodies against the TREK-1 protein (F6, 1:300; Santa Cruz Biotechnology, Dallas, TX. USA). The next day, sections were rinsed three times with TBST for 5 min each time, and then incubated for 2 h at RT with a secondary antibody conjugated with Alexafluor 488-labeled anti-mouse IgG (1:500; Fisher Scientific Co, Waltham, MA. USA) in CBS. After incubation with secondary antibody, sections were washed three times with TBST for 5 min each, rinsed with TBS and counterstained with 200 nM 4′6-diamidino-2-phenylindoledihydrochloride (DAPI, Fisher Scientific Co, Waltham, MA. USA) in TBS for 3–5 min at RT and rinsed twice in TBS. Coverslips were mounted with Fluoromount-G (SoutherBiotech, Birmingham, AL. USA). Imaging was performed on an Olympus FV1000 confocal microscope with 40X plan-apo/1.4 numerical aperture objective and FV-viewer software (Olympus, Tokyo, JP). For visualization, three-dimensional z-stack images of x-y sections at 0.5 μm steps were collected. Two-dimensional average intensity projection images were generated for analysis in FIJI (Fiji Is Image J, [50].

### In vitro studies of bladder smooth muscle contractile function

Bladder smooth muscle (BSM) strips were isolated from WT (*N* = 17) and TREK-1 KO (N = 17) mice following the previously described procedures [23, 34]. Briefly, BSM strips (∼8–10 mg each, 2–3 mm wide and 7–8 mm long) were placed in individual organ baths (Radnoti LLC, Monrovia, CA, USA), containing 7 ml of normal Tyrode Buffer (TB, in mM: NaCl 130.0, KCl 5.0, CaCl_2_ 1.7, MgCl_2_ 1.0, NaH_2_PO_4_ 1.3, NaHCO_3_ 17.0, Glucose, 10.0. pH 7.4) maintained at 37 °C and equilibrated with a constant supply of 95% O_2_–5% CO_2_. Strips were subjected to 1 h equilibration followed by determination of the length of optimal force development (*L*_o_). 1 μM of tetrodotoxin (TTX, Sigma-Aldrich) was added to fresh TB to minimize neural effects on BSM contractility. The strips were subjected to different pharmacological treatments including either arachidonic acid (AA; 10 μM), a TREK-1 channel agonist, or L-methionine (LM, 1 mM), a TREK-1 channel blocker [5, 34, 42]. Untreated muscle strips served as controls. At the end of the incubation period, in order to assess the effects of TREK-1 channels on stress-relaxation, all strips were subjected to an additional 30% stretch to the initially established *L*_o,_ and allowed for complete relaxation. Following the stretch protocol, a KCl test (bath solution was replaced with TB containing 125 mM KCl) was performed to ensure that the BSM could maintain the force after stretch [23, 24]. After KCl stimulation, muscle strips were washed three times (10 min each time) in fresh TB and allowed to recover for 30 min. After recovery, all strips were incubated with a non-specific PKC activator, Phorbol 12,13-Dibutyrate (PDBu, 1 μM: Sigma; Greenwood Village, CO), for 45 min.

A separate set of experiments was performed to evaluate the contribution of active and passive basal tone components in BSM from WT and TREK-1 KO bladders. After establishing L_0_ in normal Ca^2+^ TB, the buffer was changed to Ca^2+^ free TB followed by 30 min incubation and measurements of the basal tone and spontaneous contractile activity of BSM strips. Some strips were subjected to an additional 30% stretch to evaluate stress-relaxation under Ca^2^ free conditions. At the end of the testing protocol, all strips were treated with 2 μM of nifedipine, an L-type Ca^2+^ channel blocker, to assess the role of Ca^2+^ entry via voltage-gated Ca^2+^ channels on BSM tone and spontaneous activity.

All BSM experiments were recorded and analyzed using LabChart 8 Pro (AD Instruments, Colorado Springs, CO, USA), pClamp 10 (Molecular Devices, LLC. San Jose CA, USA) and Matlab R2014b (MathWorks, Natick, MA, USA). The following parameters were measured and analyzed: basal muscle tone (in g/g, g of force per g of muscle strip weight), amplitude of spontaneous contractions (g/g, measured as amplitude from the baseline level of each strip and normalized to its weight), peak force (PF; in g or g/g when normalized to the weight of the muscle strip), and integral force (IF) which reflects how long a muscle strip can maintain the contractile force before it starts relaxing [33].

### Urodynamic evaluation of bladder function (awake cystometry)

Animals assigned for urodynamic evaluation of urinary bladder function (awake cystometry) underwent a survival surgical procedure to insert bladder catheters as previously described [35]. Briefly, the animals were anesthetized with isofluorane (VEDCO, St.. Albans, VT) and the bladder was exposed through a lower midline abdominal incision. A polyethylene tubing (PE-50, I.D. 0.58 mm, O.D. 0.96 mm; Intramedic, Becton Dickinson. Parsippany, NY) with a flared end was inserted through a puncture at the bladder dome, and sutured in place with purse string suture and 7.0 silk (Ethicon, Somerville, NJ). The catheter was then tunneled subcutaneously and exteriorized at the scapular region where it was sutured to the skin and filled with sterile saline solution. After confirming no leakage in the bladder, the catheter was plugged with a metal rod, and the muscle and skin layers were closed with a 5.0 silk suture (Ethicon, Somerville, NJ). Particular care was taken not to stretch the bladder during the procedure or to restrict the normal bladder movement once the catheter was in place.

Mice were allowed to recover from surgery (4–5 days) before the urodynamic study. Unanesthetized animals were placed in the cystometry cage, and allowed to acclimate for 30–40 min. Saline was slowly infused into the urinary bladder at a rate of 10 μl/min, and micturition cycles were recorded using the MED-CMG Small Animal Cystometry acquisition software (Catamount Research and Development). The following cystometric parameters were evaluated in this study: bladder capacity, pressure at the start of micturition, micturition rate, intravesical pressure, inter-micturition interval, and number of non-voiding contractions. Non-voiding contractions were defined as the increased values in detrusor pressure not associated with voiding, equal or larger than twice the mean value of baseline. The number of non-voiding contractions was measured for each micturition cycle, and then averaged per cycle based on the number of recorded cycles (e.g. voiding episodes) per animal. Normal voiding contractions had amplitudes of at least a third of maximal pressure recorded during a single micturition event.

### Statistical analyses

The results are expressed as the mean ± standard error of the mean (SEM) with N reflecting the number of animals in each group and n being the number of recordings. Statistical significance between two groups was assessed by the Student two-way t-test followed by a comparison between groups using the Bonferroni’s correction. When comparing more than two groups, one-way ANOVA with the post hoc Newman-Keuls test was reported (Prism 7. GraphPad Software, La Jolla, CA. USA). Plots were made with OriginLab Data analysis software v 7.0 (OriginLab Co. Northampton, MA). Difference between the groups and treatments was considered statistically significant at *p* ≤ 0.05.

## Results

### Genotyping of TREK-1 KO mice

**Fig. 1:**
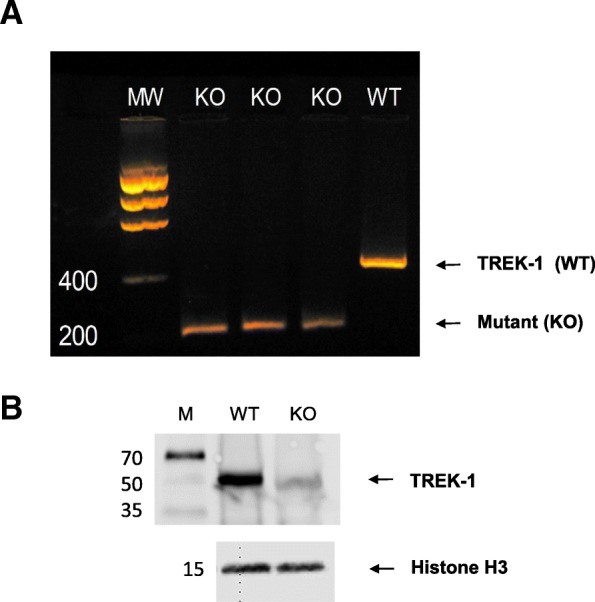
Genotyping of WT and TREK-1 KO mice. **a** Representative agarose gel showing the amplicons of wild-type (C57BL/6 J, WT, *N* = 1) and TREK-1 KO (KO, *N* = 3) alleles. The first column represents the molecular weight marker (MW) with the following three lines corresponding to the amplicon products generated by KO mRNA. The fourth line illustrates the amplicon produced by WT mRNA for comparison. The size of the bands corresponding to the TREK-1 KO (KO) and WT alleles are around ~ 200 and ~ 450 bp, respectively, as previously reported [45]. **b** Western blotting of total bladder protein from WT and TREK-1 KO animals showing a prominent band at ~ 50–52 kDa in WT mice but not in TREK-1 KO group (KO). Histone H3 was used as loading control

In TREK-1 KO mice used for this study, the four transmembrane domains and two-pore forming regions were genetically truncated by replacing most of exon 2 and all exon 3 by a LacZ/Neo cassette [45]. We confirmed the deletions by performing end-point RT-PCR in mRNA extracted from TREK-1 KO and WT bladder tissue samples (*N* = 5 in each group). The WT primers were designed, as previously described, to include parts of intron 2 and exon 3 sequences, while the KO primers included part of the LacZ/Neo cassette and intron 3 [14]. The results of RT-PCR genotyping from TREK-1 KO and WT mice are shown in Fig. 1a. The sizes of the bands corresponding to TREK-1 KO and WT alleles were around ~ 200 and ~ 450 bp, respectively. Figure 1b represents a Western Blot image of the total protein isolated from WT, and TREK-1 KO mouse bladders probed with an anti-TREK-1 antibody showing one band ~ 50 kDa in WT but not in TREK-1 KO specimens (N = 5 in each group). As previously reported [14, 45], no significative changes were observed in fertility, number of the offspring or growth rate between the WT and TREK-1 KO groups.

### Expression of TREK-1 in the visceral organs of WT and TREK-1 KO mice

**Fig. 2:**
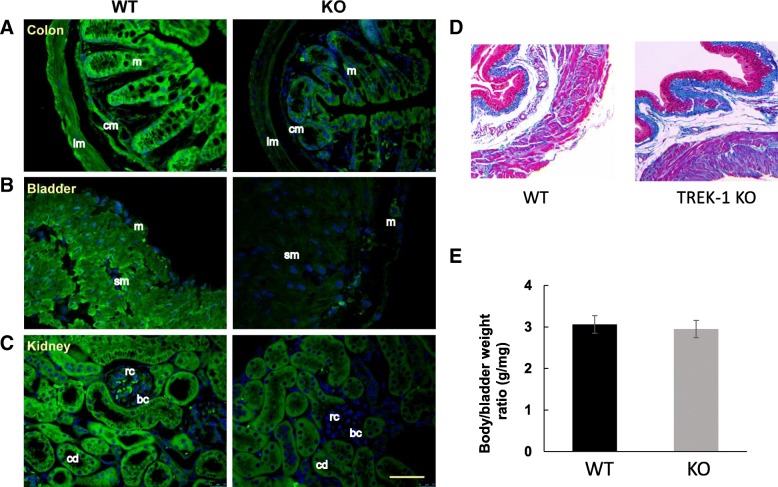
Comparison of morphological phenotypes and spatial distribution of TREK-1 channel in visceral organs of WT and TREK-1 KO mice. Representative images of immunohistochemically labeled TREK-1 protein in paraffin sections obtained from WT (left panels, *N* = 4, *n* = 20) and TREK-1 KO (right panels, *N* = 4, *n* = 20) mice. **a)** Colon, **b)** Urinary bladder, **c)** Kidneys. Used abbreviations: m-mucosa; cm-circular muscle; lm-longitudinal muscle; sm-smooth muscle; rc-renal corpuscle; cd-collecting ducts; bc-Bowman’s capsule. The scale bar is 50 μm. **d** Trichrome staining of urinary bladder cross-sections from WT and TREK-1 KO mice (10x). E) Body/bladder weight ratio in WT and TREK-1 KO animals

Immunofluorescent tissue labeling was used to compare the presence and distribution of TREK-1 proteins in the visceral organs of WT and TREK-1 KO mice including the urinary bladder, colon, and kidneys. As shown in Fig. 2a (left panel), TREK-1 was expressed in the colon of WT animals with stronger labeling present in the colonic mucosa in comparison to the muscle staining. Intense TREK-1 immunoreactivity was also found in the muscularis mucosa layer. In contrast, TREK-1 KO animals showed a significantly reduced TREK-1 immunoreactivity across the colonic tissue (Fig. 2a, right panel). Confocal images of the bladder sections from WT group (Fig. 2b, left panel) displayed a homogeneous TREK-1 like staining across the detrusor with faint signal in the urothelium and serosa. In contrast, TREK-1 KO animals showed a significant reduction in TREK-1 immunofluorescence. Histologically, the urinary bladders showed no visible morphological alterations (Fig. 2d), and no significant differences were observed in bladder weight between WT and TREK-1 KO animals (9.3 ± 0.7 vs. 10.2 ± 0.6 mg, respectively). The body/bladder weight ratio (g/mg) in WT and TREK-1 KO mice was also similar between the groups (Fig. 2e). The kidneys were bean-shaped, ~ 1.0 cm in length and weighted individually 0.50 ± 0.1 g and 0.46 ± 0.1 g (WT and TREK-1 KO groups, respectively). Histological images taken at the cortex level showed no visible histological changes in TREK-1 KO mice compared with WT aminals. In WT mice, most of the tubules in the kidney nephrons were positively labeled with TREK-1 antibody (Fig. 2c, left panel) with very faint or no positive reaction at the glomerular level. A substantial reduction in TREK-1 immunoreactivity was observed in the kidneys from TREK-1 KO animals (Fig. 2c, right panel).

### Deficiency of TREK-1 channel is associated with increased basal muscle tone and spontaneous contractions in the detrusor

**Fig. 3:**
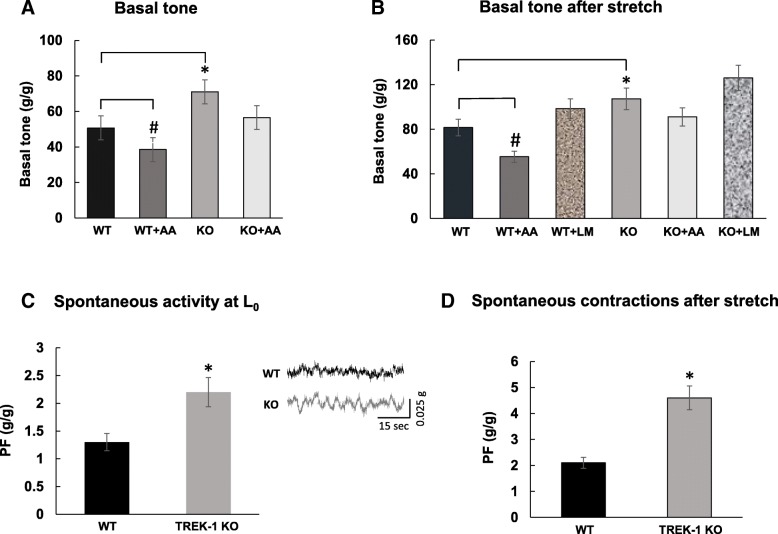
Increased basal tone and amplitude of spontaneous contractions in the detrusor of TREK-1 KO mice. **a** The basal tone of BSM strips isolated from WT (*N* = 17, *n* = 34) and TREK-1 KO (*N* = 17, *n* = 34, KO) mice was measured at L_0._ Incubation with arachidonic acid (AA) significantly reduced the basal tone in WT (WT + AA, N = 4, *n* = 8, *p* ≤ 0.05) but not in TREK-1 KO group. **b** Application of additional stretch (30% to L_0_) caused an increase in basal tone of both WT and KO strips. No significant changes in response to TREK-1 inhibitor, L-methionine (LM), were recorded in both groups. **c** The amplitude of spontaneous contractions at L_0_ was significantly elevated in TREK-1 KO group in comparison to WT group. The insert shows zoomed-in raw traces from both experimental groups. **d** Stretch protocol increased the amplitude of spontaneous contractions in both WT and TREK-1 KO BSM strips. * - *p* ≤ 0.05 to respective WT group, # - *p* ≤ 0.05 within the group

We next evaluated the role of TREK-1 channels in the maintenance of the basal smooth muscle tone and spontaneous contractions in vitro*.* The weight of BSM strips isolated from WT and TREK-1 KO mice and used for contractile function studies was similar between the groups (8.0 ± 1.5 mg and 9.1 ± 2.1 mg, respectively, *N* = 17 and *n* = 34 in each group). The basal muscle tone of the strips at their optimal length (L0) was 50.7 ± 6.1 g/g in WT and 71.2 ± 8.5 g/g in TREK-1 KO mice (Fig. 3 a, *N* = 17; n = 34 in each group, *p ≤ 0.01).* Application of AA caused 24% reduction in basal tone of WT muscle strips (from 50.7 ± 6.1 g/g to 38.5 ± 4.6 g/g, *p = 0.025*) whereas the strips from TREK-1 KO bladders had a similar tendency in response to AA, however, did not reach a level of statistical significance (Fig. 3a). In a different set of experiments, BSM strips underwent 30% stretch in addition to L_0_ in order to evaluate stress-relaxation of the detrusor when the bladder undergoes a significant stretch upon reaching its maximal capacity. After the stretch protocol, BSM strips from WT mice experienced an increase in the basal tone up to 81.5 ± 7.3 g/g (58.3% to L_0_ level, *p ≤ 0.05*, Fig. 3b), whereas the strips from TREK-1 KO mice had an increase of 50% (from 71.2 ± 8.5 g/g to 107.2 ± 9.6 g/g, *p ≤ 0.05* to L_0_ level*).* Application of LM did not significantly affect the basal muscle tone of the stretched strips in either group, however, incubation with AA led to 32% decrease in WT group (*N* = 8, *n* = 8, *p ≤ 0.05* to WT) without causing significant changes in TREK-1 KO group (N = 8, *n* = 9, Fig. 3 b). Analysis of spontaneous activity of BSM strips at L_0_ revealed the presence of spontaneous contractions in TREK-1 KO group (2.2 ± 0.2 g/g, Fig. 3c*, p ≤ 0.05* to WT) in comparison to lower contractile activity in WT group (1.3 ± 0.1 g/g, Fig. 3 c, an insert shows representative raw traces of the spontaneous activity in both groups). Additional stretch increased the amplitude of spontaneous contractions by 61% in WT group (Fig. 3d, *p* ≤ 0.05 to respective L_0_ level) with the amplitude of spontaneous contractions in BSM strips being doubled in TREK-1 KO group (109% increase to respective L_0_, Fig. 3d).

**Fig. 4:**
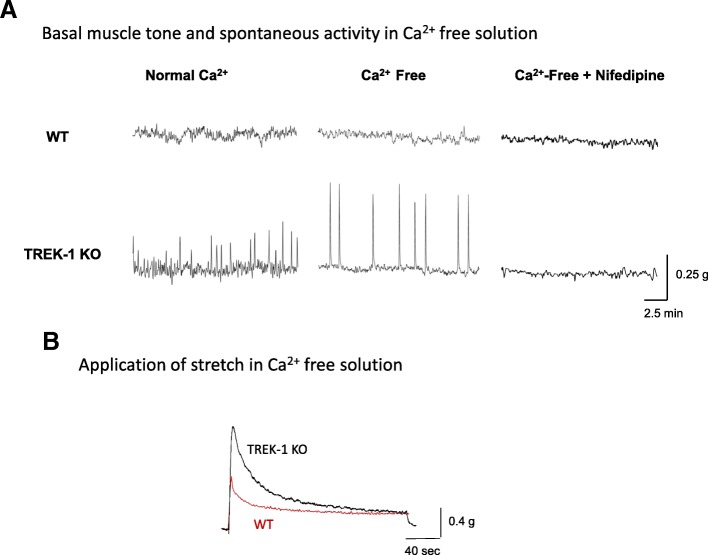
The effects of Ca^2+^ free solution and nifedipine on the basal muscle tone and spontaneous contractions in TREK-1 KO mice. **a** Representative traces of muscle tone and spontaneous activity in BSM strips from WT (upper panels) and TREK-1 KO (lower panels) animals. **b** Application of additional stretch under Ca^2+^ free conditions reduced the amplitude of the contractile response in both WT and TREK-1 KO animals

The differences observed in the basal muscle tone and frequency of spontaneous activity between WT and TREK-1 KO BSM strips were further tested in Ca^2+^ free TB to evaluate the role of active contractile processes due to Ca^2+^ influx into the cells. First, we incubated BSM from WT and TREK-1 KO animals in Ca-free solution for 30 min followed by either 30% additional stretch or incubation with nifedipine, a general L-type calcium channel blocker (2 μM, 30 min). Representitave recordings from WT and TREK-1 KO BSM strips are included in Fig. 4. Substitution of normal TB by Ca^2+^-free TB reduced the basal muscle tone in both WT and TREK-1 KO BMS strips when compared to normal Ca^2+^ solution (Fig. 4a, middle traces). However, BSM from TREK-1 KO mice showed a ~ 2.0 fold increase in the amplitude of spontaneous contractions (from 2.2 ± 0.1 g/g in normal Ca^2+^ to 4.1 ± 0.1 g/g in Ca^2+^-free TB, *N* = 4,*n* = 8 in each group, *p* ≤ 0.05 to normal Ca TB). Application of additional stretch under Ca^2+^ free conditions showed decreased values of PF in both WT and TREK-1 KO groups by ~ 30–32% when compared to normal Ca^2+^ solution. The contractile reponses also had a significantly faster initial decline phase of stress-relaxation in both groups (Fig. 4b).

### Enhanced force generation to electric field stimulation and high potassium in BSM strips from TREK-1 KO mice

**Fig. 5:**
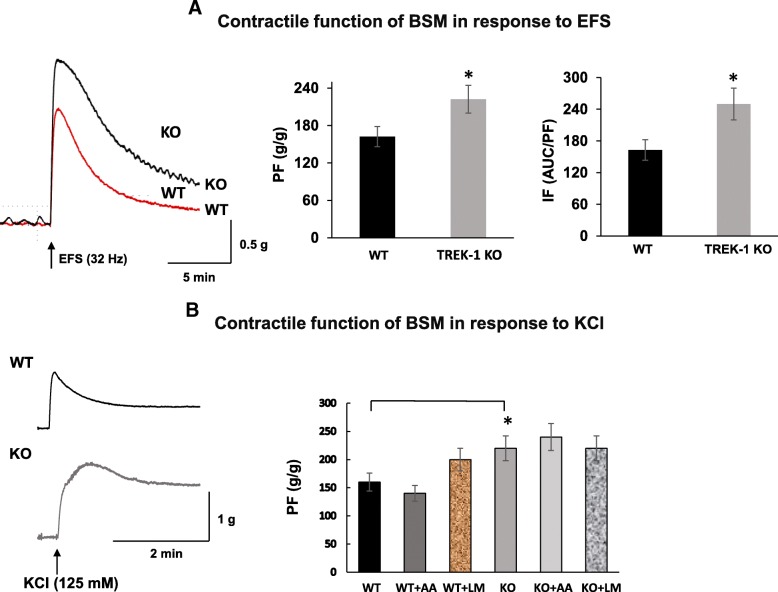
Effect of electric field stimulation and high potassium on WT and TREK-1 KO bladder detrusor strip contractile function. **a** Isolated BSM strips from TREK-1 KO mice showed significantly higher contractile force in response to EFS (80 V, 32 Hz, 1 ms duration). The left panel shows overlapped examples of raw traces from both groups. Middle panel represents the summary of PF normalized to the weight of tissue strips whereas right panel shows the integral force for both groups. **b** Effect of KCl stimulation on PF in WT (*N* = 17, *n* = 34) and TREK-1 KO (*N* = 17, *n* = 34) groups before and after pharmacological activation (by AA) and inhibition (by ML) of TREK-1 channels. Left panel represents raw traces of recordings. * - *p* ≤ 0.05 to WT group

Bladder smooth muscle strips from TREK-1 KO mice generated more contractile force in response to EFS (32 Hz) in comparison to WT group (222.2 ± 21.2 g/g and 162.2 ± 16.2 g/g, *p ≤ 0.05*, Fig. 5a, middle panel). The IF calculated as ratio of the area under the curve (AUC) of a single contraction divided by its PF was also significantly higher for TREK-1 KO group in comparison to WT (250 ± 30.1 vs 163.1 ± 19.6*, p ≤ 0.05*, Fig. 5a, right panel). Similar to the effects of EFS, TREK-1 KO strips also revealed an increased contractile response to high KCl stimulation (Fig. 5b). However, application of TREK-1 inhibitor (LM) and activator (AA) did not substantially change the contractile responses to high potassium in both WT and TREK-1 KO groups in comparison to baseline values (Fig. 5b).

**Fig. 6:**
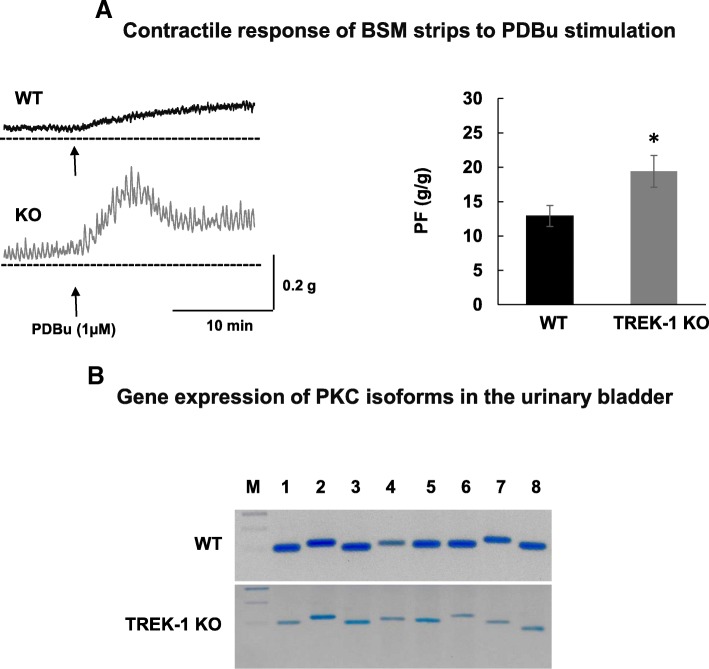
Activation of PKC pathway in the urinary bladder is associated with higher response to stimulation in TREK-1 KO mice. **a** Effects of PKC activator, PDBu (1 μM) on contractile function of WT (*N* = 8, *n* = 8) and TREK-1 KO (*N* = 8, *n* = 9, left panel) muscle strips. Right panel indicates that PDBu induced a significant increase in PF in TREK-1 KO group when compared to WT bladders. **b** RT-PCR results show gene expression of 7 major PKC isoforms expressed in the urinary bladders from both WT and TREK-1 KO mice. Bands from 1 to 7 represent the following PKC isoforms: 1 - PKC-α (alpha), 2 - PKC-β (beta), 3 - PKC-γ (gamma), 4 - PKC- δ (delta), 5 - PKC-ε (epsilon), 6 – PKC- μ (mu), 7- PKC-τ (tau). Line 8 - GAPDH. M- marker, * - *p* ≤ 0.05 to WT group

Previous studies from our laboratory established that PKC activation with general agonist phorbol-12,13-dibutyrate (PDBu) significantly reduced the amplitude and increased the frequency of spontaneous BSM contractions at low concentrations (10 nM) while causing an increase in muscle force generation at higher concentrations (1 μM). Although several previous studies provided evidence that PKC mostly affects large conductance Ca^2+^-activated potassium channels (BK), TREK-1 channels could also be modulated by PKC phosphorylation. Therefore, we tested the reponses to a PKC activator in TREK-1 KO bladders. Application of PDBu (1 μM) significantly increased PF (by 50%, *p ≤ 0.05*, Fig. 6a) and the amplitude of spontaneous contractions in TREK-1 KO muscle strips when compared to WT group. Protein kinase C conveys both calcium-dependent, and calcium-independent effects on DSM contractility in vitro mediated via inhibition of myosine light chain phosphatase. However, it is still unknown if these separate effects are mediated by different PKC isoforms. Therefore, we aimed to test if elevated reponses to PKC stimulation in TREK-1 KO bladders may be due to compensatory changes in expression levels of the PKC isoforms present in the detrusor. The results of RT-PCR presented in Fig. 6b did not reveal any significant changes in the expression levels of 7 tested PKC isoforms including (PKC-α (alpha), PKC-β (beta), PKC-γ (gamma), PKC- δ (delta), PKC-ε (epsilon), PKC-μ (mu) and PKC-τ (tau)) between the WT and TREK-1 groups. This data suggests that the effects are likely due to the secondary regulatory changes in PKC–related signaling pathways.

### Urodynamic analysis of bladder function in TREK-1 KO mice

**Fig. 7:**
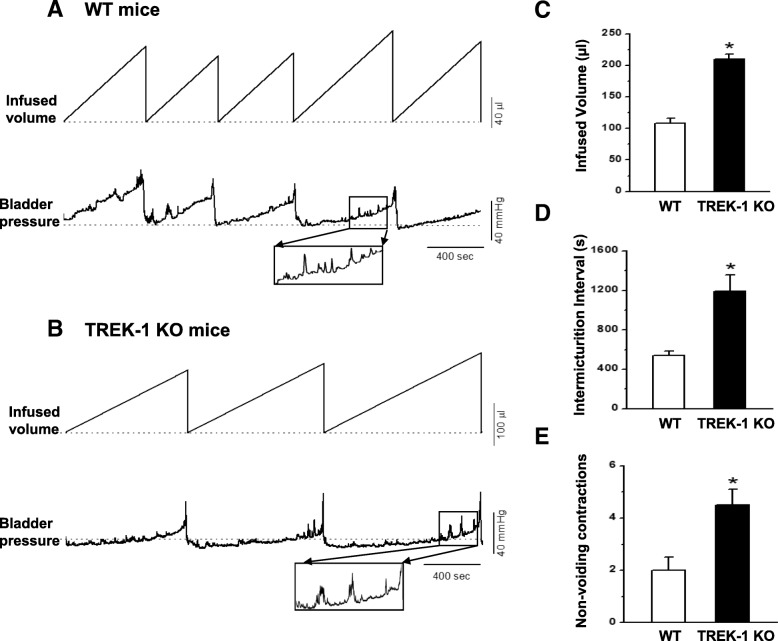
Urodynamic evaluation of bladder function in WT and TREK-1 KO mice. **a** Representative cystometrogram traces recorded in freely moving WT (upper panels) and TREK-1 KO (bottom panels) mice. The volume of the infused saline and bladder pressure are included in the traces. **b** Analysis of bladder capacity (BC) in WT and TREK-1 KO groups. **c** Comparison of the total volume infused before micturition in WT and TREK-KO mice. **d** Duration of inter-micturition interval in WT and genetically modified mice. **e** The average number of non-voiding contractions (per micturition cycle) recorded in WT and TREK-1 KO mice. Zoomed-in segments of the traces indicated by squares are shown in the inserts. * - significance level of *p* ≤ 0.01 to WT group

The analysis of cystometrograms recorded under control conditions in WT (*N* = 5) and TREK-1 KO (*N* = 6) mice showed an approximately 2-fold increase in bladder capacity in TREK-1 KO animals in comparison to WT mice (210.0 ± 8.1 μl vs. 108.2 ± 8.0 μl, respectively.Figure 7 a, b and c. *p ≤ 0.05*), and a significant prolongation of the intermicturition interval (540.0 ± 43.8 s vs 1188.4 ± 166.4 s, WT and KO mice, respectively. Figure 7d. *p ≤ 0.05*). The substantially elevated volume of infused saline during storage phase, which is reflective of increased bladder capacity, with the prolonged duration of the voiding cycle point toward the development of an underactive phenotype in TREK-1 KO mice. However, although no differences were observed in bladder pressure at voiding (32.1 ± 3.2 mmHg vs. 39.0 ± 2.7 mmHg, TREK-1 KO, and WT mice, respectively), BMS from TREK-1 KO animals showed a 2-fold increase in the number of non-voiding contractions (TREK-1 KO: 4.5 ± 0.6 vs WT: 2.0 ± 0.5, *p ≤ 0.05*, Fig. 7e) per micturition cycle. Other urodynamic characteristics were not different between the WT and TREK-1 KO animals. Therefore, the overall TREK-1 KO bladder phenotype displays the characteristics of both bladder under- and overactivity.

## Discussion

Evaluation of bladder phenotype in TREK-1 KO animals revealed significant differences in comparison to WT mice including elevated basal muscle tone, increased amplitude of basal spontaneous activity, limited pharmacological responses to TREK-1 activators and inhibitors, and elevated contractile responses to EFS and high potassium without significant changes in bladder morphology/histology. However, urodynamic study in TREK-1 KO mice revealed a substantially longer inter-micturition interval, enhanced bladder capacity and increased number of non-voiding contractions in comparison to WT animals. Overall, the results of the combined in vivo and in vitro experiments provided evidence that global down-regulation of TREK-1 channel leads to “mixed” voiding phenotype in TREK-1 KO mice.

TREK-1 channel belongs to a family of mechanogated two-pore domain potassium channels (K_2P_) that produce background conductances, and are regulated by a variety of stimuli (e.g., pH, temperature, stretch and lipids) to control resting membrane potential and cell excitability [7]. The TREK subfamily, encoded by the *KCNK2* gene, consists of three known members (TREK-1, TREK-2 and TRAAK) which participate in diverse transduction processes including mechano-sensitivity [6, 36, 38, 47], thermo-sensitivity [2, 26, 39], chemo-sensitivity [40], nociception, and neuroprotection [12, 15, 20, 31].

The results of Western blotting and IHC demonstrated that TREK-1 protein was substantially diminished in the urinary bladder of TREK-1 KO mice. The antibodies used for WB and IHC should detect any TREK-1 protein being translated, regardless of which one of the multiple start codons was used to initiate translation. Since we used the TREK-1 KO strain created by Namiranian et al. [45] in which both the second and third exons of *Kcnk2* gene were deleted, all start codons present in both exons were deleted in this strain. Another group [17] created TREK-1 KO strain in which only the third exon was removed leaving an alternative start codon in exon 2, and, therefore, a truncated TREK-1 protein could be translated. In our study, we tested several TREK-1 antibodies. The anti-TREK-1 mouse monoclonal antibody from SantaCruz Biotechnology (F6, sc-398,449; mapped at aa354–380 near the C-terminus of hTREK-1) showed only one band ~ 50–52 kDa when used in WB (Fig. 1b). We also tested other commercially available antibodies from the same company which included the C20, E-19, and H-75 anti-TREK-1 variants. The C20 and E19 anti-TREK-1 antibodies did not work for Western blotting, while H-75 antibody (aa352–426 within the C-terminus) showed two bands in WT animals and one band in TREK-1 KO (data not shown) suggesting that it might be recognizing alternatively spliced variants or “off-target” proteins.

Increased basal muscle tone in the bladder of TREK-1 KO mice supports previous suggestions about the role of TREK-1 channel in maintaining the negative resting membrane potential in visceral smooth muscle [45]. Bladder muscle strips from TREK-1 KO mice also revealed the presence of spontaneous activity at both resting level and after additional stretch. Spontaneous contractions have been previously recorded in a variety of bladder smooth muscle types including human [21, 58], rabbit [11, 24], rat [23], guinea pig [56], pig [25, 56], and mouse [28, 54]. It is possible that knockdown of TREK-1 may affect calcium mobilization and modify the threshold for smooth muscle excitability and/or contractility, thereby, increasing spontaneous activity, as observed in our study. To test this hypothesis, we incubated BMS strips in Ca^2+^-free TB followed by the application of nifedipine, a L-type calcium channel blocker (Fig. 4). Incubation of TREK-1 KO strips in Ca^2+^ − free solution effectively reduced the basal tone to the levels similar to those observed in WT BMS strips. Interestingly, the reduced muscle tone observed in Ca^2+^-free solution was associated with a 2-fold increase in the amplitude of spontanous contractions in TREK-1 KO group. The total elimination of spontaneous contractions and further reduction in basal tone by nifedipine suggests that TREK-1 channels participate in regulation of spontaneous contractions by stabilizing the membrane potential at voltages closer to the potassium resting potential. Overall, in addition to previously published data on human [3, 34] and animal [22, 42] visceral smooth muscle, our data provide additional evidence that TREK-1 channel plays a critical role in maintaining bladder basal tone, bladder compliance and modulates the response of the detrusor to stretch.

The contractile responses of BSM to EFS are mainly mediated by the release of neurotransmitters from intramural nerve terminals in the bladder wall causing detrusor contraction. We compared the responses of TREK-1 KO and WT muscle strips in their ability to generate force in response to EFS. The data showed that the loss of TREK-1 channels was associated with a significant increase in peak contractility in response to EFS (Fig. 5) while slowing the rate of relaxation in the TREK-1 KO muscle strips when compared to WT group. While EFS induces a contraction mainly via the release of acetylcholine from bladder nerves, KCl-induced contractions result from direct activation and depolarization of the detrusor muscle due to rapid membrane depolarization and the associated influx of Ca^2+^ via voltage-gated Ca^2+^ channels. Similar to EFS, high potassium solution also caused a significantly elevated amplitude of the contractile response in TREK-1 KO strips suggesting that increased contractility in the absence of TREK-1 channels is due to a partial membrane depolarization which shifts the resting membrane potential towards more positive values closer to the activation threshold of voltage-gated calcium channels.

Prior studies have reported that protein kinase C (PKC) may be able to phosphorylate TREK-1 channels in smooth muscle, thereby, modulating the contractile force [30, 37, 43]. We previously showed that activation of PKC by general PKC activator, PDBu, at low levels of stimulation could inhibit spontaneous contractions in rabbit [24] and rat [23] BSM while higher levels of PKC stimulation had the opposite effect. These data predict that upon down-regulation of TREK-1, the PKC activator, PDBu, should have a more positive contractile effect on KO muscle strips when compared to WT group. Our data are consistent with this analysis in that PDBu, indeed, increased both the PF and the amplitude of spontaneous contractions in TREK-1 KO muscle strips compared to the WT mice. Analysis of gene expression of different PKC isoforms expressed in the urinary bladder did not reveal significant differences between WT and TREK-1 KO mice. Therefore, additional studies will be required to evaluate if the elevated response to PKC activation in TREK-1 KO bladder is due to the changes in PKC phosphorylation or activation of different downstream signaling pathways.

In contrast to in vitro contractility results, the urodynamic evaluation of voiding function in vivo by awake cystometry revealed a significant increase in the duration of inter-micturition interval and enhanced bladder capacity, both of which usually define an underactive bladder phenotype. Despite these changes, the intravesical pressure during micturition was not affected by the TREK-1 knockdown, and the number of non-voiding contractions was almost 2-fold higher in these animals which is in line with the increased contractility of the detrusor observed during the in vitro studies discussed above. Taken together, these results suggest that knock-down of *Kcnk2* gene has dual or “mixed” effects on detrusor contractility and micturition patterns. One of the possible explanations could be associated with expression of the channel on vascular and visceral smooth muscle cells as well as on the fibers and neurons of the peripheral and central nervous system [19, 41]. The functional role of TREK-1 in smooth muscle cells is mainly to contribute to the “leak” current responsible for the maintenance of hyperpolarized resting membrane potential to keep the cells more relaxed during resting state [5, 10].

In the nervous system, TREK-1 was shown to control electrogenesis, differentiation, axonal migration, synaptogenesis, and neural response to temperature and mechanical stretch [1, 9, 13, 16, 19, 27, 31, 51]. In comparison to smooth muscle cells, where action potentials are driven by the influx of Ca^2+^ via L-type Ca^2+^ channels, excitability of neurons is regulated mainly by voltage-gated Na^+^ channels. The lack of TREK-1 channel in neurons would shift the resting membrane potential towards more positive values (just like in smooth muscle cells) allowing for the rapid influx of Na^+^ inside the cell, and, therefore, making both afferent and efferent neurons more excitable. The final result of this neuronal activity would depend on what neurons are more affected by these changes. An increase in afferent activity from the urinary bladder to the brain is usually associated with an increase in urgency and frequency of micturition associated with bladder overactivity. However, the efferent output from the brain to the bladder is mainly inhibitory to allow for the voluntary control of micturition. Therefore, increased excitability of efferent neurons would lead to increased inter-micturition interval and bladder underactivity, which we observed in our study in vivo. Future studies focused on manipulations of either afferent or efferent neural pathways are warranted to test this hypothesis.

Another possible explanation for the differences between in vitro and in vivo bladder phenotypes could be associated with some unknown alterations occurring during genetic modifications of the parental strain. TREK-1 was determined to mediate changes in the actin cytoskeleton independently of its channel activity in fetal neurons during development in a different strain of TREK-1 KO mice [32]. The same mouse strain showed an increased efficacy of 5-HT neurotransmission and resistance to depression [20]. Previous studies from our [35] and other [57] groups provided evidence that lower urinary tract function in mice varies in a consistent manner with strain and/or sex. For instance, voiding spot assay and cystometry performed in male and female C57BL/6 J, 129S1/SvImJ, NOD/ShiLtJ, and CAST/EiJ mice [8] to evaluate bladder function, established a significantly prolonged duration of the micturition cycle in genetically modified animals (129S1/SvImJ, NOD/ShiLtJ, and CAST/EiJ) in comparison to C57BL/6 J strain [8]. These results are in line with our cystometric recordings from TREK-1 KO mice. The differences between our study and the published results included the use of both sexes (males and females), the use of WT controls which were not littermates, and performance of cystometry without anesthesia in our experiments, as well as the absence of in vitro experiments in the previously published study.

The choice of the approach to create a genetically modified strain also seems to affect the function of different organs and tissues in genetically modified animals. For instance, the TREK-1 KO strain we adopted and used in our study, provided no evidence that TREK-1 is involved in the regulation of arterial diameter in cerebral arteries [45]. However, another TREK-1 KO strain [32] did present with the vascular phenotype. There is also a possibility that genetic knockdown of TREK-1 expression could trigger compensatory changes in other mechano-gated ion channels or related signaling cascades, thereby, affecting the final voiding phenotype as well as the response of the detrusor to stretch. Further studies are warranted to address these questions.

## Conclusions

Our study provided evidence that global down-regulation of TREK-1 channels has dual effects on detrusor contractility and micturition patterns in vivo. The integrative effects of TREK-1, likely, depend on the expression and function of the channel not only in detrusor myocytes but also in afferent and efferent neural pathways regulating micturition. Future studies are warranted to identify the precise mechanisms of TREK-1 associated mechanotransduction between detrusor myocytes and afferent nerves in the bladder wall, as well as the role of TREK-1 in efferent fibers and central nervous system centers controlling voiding. This knowledge would provide a foundation for the development of novel therapeutic approaches to treat voiding dysfunction in patients with detrusor overactivity, overactive bladder, and bladder pain syndrome.

## Abbreviations

AA: Arachidonic acid
AUC: Area under the curve
BK: Ca^2+^-activated potassium channels
BSM: Bladder smooth muscle
DO: Detrusor overactivity
EFS: Electric field stimulation
IF: Integral force
IHC: Immunohistochemistry
KO: Knockout
L_0_: The length of optimal force development
LM: L-methionine
PBS: Phosphate-buffered saline
PDBu: Phorbol 12,13-Dibutyrate
PF: Peak force
PFA: Paraformaldehyde
PKC: Protein kinase C
RT: Room temperature
TB: Tyrode Buffer
TBS: Tris-Buffered saline
TREK-1 channel: Two-pore domain potassium channel encoded by *Kcnk2* gene
TTX: Tetrodotoxin
WB: Western blotting
WT: Wild type

## References

[1] Afzali AM, Ruck T, Herrmann AM, Iking J, Sommer C, Kleinschnitz C, Preubetae C, Stenzel W, Budde T, Wiendl H, Bittner S, Meuth SG (2016). The potassium channels TASK2 and TREK1 regulate functional differentiation of murine skeletal muscle cells. Am J Physiol Cell Physiol.

[2] Alloui A, Zimmermann K, Mamet J, Duprat F, Noel J, Chemin J, Guy N, Blondeau N, Voilley N, Rubat-Coudert C, Borsotto M, Romey G, Heurteaux C, Reeh P, Eschalier A, Lazdunski M (2006). TREK-1, a K+ channel involved in polymodal pain perception. EMBO J.

[3] Bai X, Bugg GJ, Greenwood SL, Glazier JD, Sibley CP, Baker PN, Taggart MJ, Fyfe GK (2005). Expression of TASK and TREK, two-pore domain K+ channels, in human myometrium. Reproduction.

[4] Baker SA, Hatton WJ, Han J, Hennig GW, Britton FC, Koh SD (2010). Role of TREK-1 potassium channel in bladder overactivity after partial bladder outlet obstruction in mouse. J Urol.

[5] Baker SA, Hennig GW, Han J, Britton FC, Smith TK, Koh SD (2008). Methionine and its derivatives increase bladder excitability by inhibiting stretch-dependent K(+) channels. Br J Pharmacol.

[6] Bang H, Kim Y, Kim D (2000). TREK-2, a new member of the mechanosensitive tandem-pore K+ channel family. J Biol Chem.

[7] Bayliss DA, Barrett PQ (2008). Emerging roles for two-pore-domain potassium channels and their potential therapeutic impact. Trends Pharmacol Sci.

[8] Bjorling DE, Wang Z, Vezina CM, Ricke WA, Keil KP, Yu W, Guo L, Zeidel ML, Hill WG (2015). Evaluation of voiding assays in mice: impact of genetic strains and sex. Am J Physiol Renal Physiol.

[9] Bockenhauer D, Zilberberg N, Goldstein SA (2001). KCNK2: reversible conversion of a hippocampal potassium leak into a voltage-dependent channel. Nat Neurosci.

[10] Buxton IL, Singer CA, Tichenor JN (2010). Expression of stretch-activated two-pore potassium channels in human myometrium in pregnancy and labor. PLoS One.

[11] Chang S, Hypolite JA, Mohanan S, Zderic SA, Wein AJ, Chacko S (2009). Alteration of the PKC-mediated signaling pathway for smooth muscle contraction in obstruction-induced hypertrophy of the urinary bladder. Lab Investig.

[12] Chemin J, Patel AJ, Duprat F, Lauritzen I, Lazdunski M, Honore E (2005). A phospholipid sensor controls mechanogating of the K+ channel TREK-1. EMBO J.

[13] Devader C, Khayachi A, Veyssiere J, Moha Ou Maati H, Roulot M, Moreno S, Borsotto M, Martin S, Heurteaux C, Mazella J (2015). In vitro and in vivo regulation of synaptogenesis by the novel antidepressant spadin. Br J Pharmacol.

[14] Du Y, Kiyoshi CM, Wang Q, Wang W, Ma B, Alford CC, Zhong S, Wan Q, Chen H, Lloyd EE, Bryan RM Jr, Zhou M. Genetic deletion of TREK-1 or TWIK-1/TREK-1 potassium channels does not Alter the basic electrophysiological properties of mature hippocampal astrocytes in situ. Front Cell Neurosci. 2016;10(13). 10.3389/fncel.2016.00013 PMC4738265 26869883

[15] Duprat F, Lesage F, Patel AJ, Fink M, Romey G, Lazdunski M (2000). The neuroprotective agent riluzole activates the two P domain K(+) channels TREK-1 and TRAAK. Mol Pharmacol.

[16] Fink M, Duprat F, Lesage F, Reyes R, Romey G, Heurteaux C, Lazdunski M (1996). Cloning, functional expression and brain localization of a novel unconventional outward rectifier K+ channel. EMBO J.

[17] Guyon A, Tardy MP, Rovere C, Nahon JL, Barhanin J, Lesage F (2009). Glucose inhibition persists in hypothalamic neurons lacking tandem-pore K+ channels. J Neurosci.

[18] Hervieu GJ, Cluderay JE, Gray CW, Green PJ, Ranson JL, Randall AD, Meadows HJ (2001). Distribution and expression of TREK-1, a two-pore-domain potassium channel, in the adult rat CNS. Neuroscience.

[19] Heurteaux C, Guy N, Laigle C, Blondeau N, Duprat F, Mazzuca M, Lang-Lazdunski L, Widmann C, Zanzouri M, Romey G, Lazdunski M (2004). TREK-1, a K+ channel involved in neuroprotection and general anesthesia. EMBO J.

[20] Heurteaux C, Lucas G, Guy N, El Yacoubi M, Thummler S, Peng XD, Noble F, Blondeau N, Widmann C, Borsotto M, Gobbi G, Vaugeois JM, Debonnel G, Lazdunski M (2006). Deletion of the background potassium channel TREK-1 results in a depression-resistant phenotype. Nat Neurosci.

[21] Hristov KL, Afeli SA, Parajuli SP, Cheng Q, Rovner ES, Petkov GV (2013). Neurogenic detrusor overactivity is associated with decreased expression and function of the large conductance voltage- and ca(2+)-activated K(+) channels. PLoS One.

[22] Hwang SJ, O'Kane N, Singer C, Ward SM, Sanders KM, Koh SD (2008). Block of inhibitory junction potentials and TREK-1 channels in murine colon by Ca2+ store-active drugs. J Physiol.

[23] Hypolite JA, Chang S, Wein AJ, Chacko S, Malykhina AP. Protein kinase C modulates frequency of micturition and non-voiding contractions in the urinary bladder via neuronal and myogenic mechanisms. BMC Urol. 2015;15(34). 10.1186/s12894-015-0030-9 PMC4407874 25896919

[24] Hypolite JA, Lei Q, Chang S, Zderic SA, Butler S, Wein AJ, Malykhina AP, Chacko S (2013). Spontaneous and evoked contractions are regulated by PKC-mediated signaling in detrusor smooth muscle: involvement of BK channels. Am J Physiol Renal Physiol.

[25] Isogai A, Lee K, Mitsui R, Hashitani H (2016). Functional coupling of TRPV4 channels and BK channels in regulating spontaneous contractions of the Guinea pig urinary bladder. Pflugers Arch.

[26] Kang D, Choe C, Kim D (2005). Thermosensitivity of the two-pore domain K+ channels TREK-2 and TRAAK. J Physiol.

[27] Kanjhan R, Anselme AM, Noakes PG, Bellingham MC (2004). Postnatal changes in TASK-1 and TREK-1 expression in rat brain stem and cerebellum. Neuroreport.

[28] Kobayter S, Young JS, Brain KL (2012). Prostaglandin E2 induces spontaneous rhythmic activity in mouse urinary bladder independently of efferent nerves. Br J Pharmacol.

[29] Koh SD, Sanders KM (2001). Stretch-dependent potassium channels in murine colonic smooth muscle cells. J Physiol.

[30] Kreneisz O, Benoit JP, Bayliss DA, Mulkey DK (2009). AMP-activated protein kinase inhibits TREK channels. J Physiol.

[31] Lauritzen I, Blondeau N, Heurteaux C, Widmann C, Romey G, Lazdunski M (2000). Polyunsaturated fatty acids are potent neuroprotectors. EMBO J.

[32] Lauritzen I, Chemin J, Honore E, Jodar M, Guy N, Lazdunski M, Jane Patel A (2005). Cross-talk between the mechano-gated K2P channel TREK-1 and the actin cytoskeleton. EMBO Rep.

[33] Lee H, Koh BH, Peri LE, Sanders KM, Koh SD (2014). Purinergic inhibitory regulation of murine detrusor muscles mediated by PDGFRalpha+ interstitial cells. J Physiol.

[34] Lei Q, Pan XQ, Chang S, Malkowicz SB, Guzzo TJ, Malykhina AP (2014). Response of the human detrusor to stretch is regulated by TREK-1, a two-pore-domain (K2P) mechano-gated potassium channel. J Physiol.

[35] Lei Q, Pan XQ, Villamor AN, Asfaw TS, Chang S, Zderic SA, Malykhina AP. Lack of transient receptor potential vanilloid 1 channel modulates the development of neurogenic bladder dysfunction induced by cross-sensitization in afferent pathways. J Neuroinflammation. 2013;10(3). 10.1186/1742-2094-10-3 PMC3556132 23305398

[36] Lesage F, Terrenoire C, Romey G, Lazdunski M (2000). Human TREK2, a 2P domain mechano-sensitive K+ channel with multiple regulations by polyunsaturated fatty acids, lysophospholipids, and Gs, Gi, and Gq protein-coupled receptors. J Biol Chem.

[37] Liu H, Enyeart JA, Enyeart JJ (2007). Angiotensin II inhibits native bTREK-1 K+ channels through a PLC-, kinase C-, and PIP2-independent pathway requiring ATP hydrolysis. Am J Physiol Cell Physiol.

[38] Maingret F, Honore E, Lazdunski M, Patel AJ (2002). Molecular basis of the voltage-dependent gating of TREK-1, a mechano-sensitive K(+) channel. Biochem Biophys Res Commun.

[39] Maingret F, Lauritzen I, Patel AJ, Heurteaux C, Reyes R, Lesage F, Lazdunski M, Honore E (2000). TREK-1 is a heat-activated background K(+) channel. EMBO J.

[40] Maingret F, Patel AJ, Lesage F, Lazdunski M, Honore E (1999). Mechano- or acid stimulation, two interactive modes of activation of the TREK-1 potassium channel. J Biol Chem.

[41] Medhurst AD, Rennie G, Chapman CG, Meadows H, Duckworth MD, Kelsell RE, Gloger II, Pangalos MN (2001). Distribution analysis of human two pore domain potassium channels in tissues of the central nervous system and periphery. Brain Res Mol Brain Res.

[42] Monaghan K, Baker SA, Dwyer L, Hatton WC, Sik Park K, Sanders KM, Koh SD (2011). The stretch-dependent potassium channel TREK-1 and its function in murine myometrium. J Physiol.

[43] Murbartian J, Lei Q, Sando JJ, Bayliss DA (2005). Sequential phosphorylation mediates receptor- and kinase-induced inhibition of TREK-1 background potassium channels. J Biol Chem.

[44] Namiranian Khodadad, Brink Christa D, Goodman Jerry Clay, Robertson Claudia S, Bryan Robert M (2010). Traumatic Brain Injury in Mice Lacking the K Channel, TREK-1. Journal of Cerebral Blood Flow & Metabolism.

[45] Namiranian Khodadad, Lloyd Eric E., Crossland Randy F., Marrelli Sean P., Taffet George E., Reddy Anilkumar K., Hartley Craig J., Bryan Robert M. (2010). Cerebrovascular responses in mice deficient in the potassium channel, TREK-1. American Journal of Physiology-Regulatory, Integrative and Comparative Physiology.

[46] Patel AJ, Honore E (2001). Properties and modulation of mammalian 2P domain K+ channels. Trends Neurosci.

[47] Patel AJ, Honore E, Maingret F, Lesage F, Fink M, Duprat F, Lazdunski M (1998). A mammalian two pore domain mechano-gated S-like K+ channel. EMBO J.

[48] Pineda RH, Nedumaran B, Hypolite J, Pan XQ, Wilson S, Meacham RB, Malykhina AP (2017). Altered expression and modulation of the two-pore-domain (K2P) mechanogated potassium channel TREK-1 in overactive human detrusor. Am J Physiol Renal Physiol.

[49] Sanders KM, Koh SD (2006). Two-pore-domain potassium channels in smooth muscles: new components of myogenic regulation. J Physiol.

[50] Schindelin J, Arganda-Carreras I, Frise E, Kaynig V, Longair M, Pietzsch T, Preibisch S, Rueden C, Saalfeld S, Schmid B, Tinevez JY, White DJ, Hartenstein V, Eliceiri K, Tomancak P, Cardona A (2012). Fiji: an open-source platform for biological-image analysis. Nat Methods.

[51] Thomas D, Plant LD, Wilkens CM, McCrossan ZA, Goldstein SA (2008). Alternative translation initiation in rat brain yields K2P2.1 potassium channels permeable to sodium. Neuron.

[52] Tichenor JN, Hansen ET, Buxton IL (2005). Expression of stretch-activated potassium channels in human myometrium. Proc West Pharmacol Soc.

[53] Wellner MC, Isenberg G (1994). Stretch effects on whole-cell currents of Guinea-pig urinary bladder myocytes. J Physiol.

[54] White RS, Zemen BG, Khan Z, Montgomery JR, Herrera GM, Meredith AL (2014). Evaluation of mouse urinary bladder smooth muscle for diurnal differences in contractile properties. Front Pharmacol.

[55] Wu YY, Singer CA, Buxton IL (2012). Variants of stretch-activated two-pore potassium channel TREK-1 associated with preterm labor in humans. Biol Reprod.

[56] Xin W, Li N, Fernandes VS, Petkov GV (2016). Constitutively active PKA regulates neuronal acetylcholine release and contractility of Guinea pig urinary bladder smooth muscle. Am J Physiol Renal Physiol.

[57] Yu W, Ackert-Bicknell C, Larigakis JD, MacIver B, Steers WD, Churchill GA, Hill WG, Zeidel ML (2014). Spontaneous voiding by mice reveals strain-specific lower urinary tract function to be a quantitative genetic trait. Am J Physiol Renal Physiol.

[58] Zagorodnyuk VP, Gregory S, Costa M, Brookes SJ, Tramontana M, Giuliani S, Maggi CA (2009). Spontaneous release of acetylcholine from autonomic nerves in the bladder. Br J Pharmacol.

[59] Pineda RH, Hypolite J, Lee S, Carrasco A, Iguchi N, Meacham RB, Malykhina AP. The lack of mechanosensitive K2P channel is associated with mixed voiding phenotype in mice. J Urology. 199(4S):e503–4.

